# Striatal dopamine receptor binding in morbidly obese women before and after gastric bypass surgery and its relationship with insulin sensitivity

**DOI:** 10.1007/s00125-014-3178-z

**Published:** 2014-02-06

**Authors:** Barbara A. de Weijer, Elsmarieke van de Giessen, Ignace Janssen, Frits J. Berends, Arnold van de Laar, Mariette T. Ackermans, Eric Fliers, Susanne E. la Fleur, Jan Booij, Mireille J. Serlie

**Affiliations:** 1 Department of Endocrinology and Metabolism, University of Amsterdam, Academic Medical Center, Meibergdreef 9, 1105 AZ Amsterdam, the Netherlands; 2 Department of Nuclear Medicine, Academic Medical Center, Amsterdam, the Netherlands; 3 Department of Surgery, Rijnstate Hospital, Arnhem, the Netherlands; 4 Department of Surgery, Slotervaart Hospital, Amsterdam, the Netherlands; 5 Department of Clinical Chemistry, Laboratory of Endocrinology, Academic Medical Center, University of Amsterdam, Amsterdam, the Netherlands

**Keywords:** Dopamine receptor, Gastric bypass surgery, Insulin sensitivity, Obesity, SPECT, Striatal dopamine, Weight loss

*To the Editor*: There is evidence that certain brain areas are functionally altered in obesity. In obese humans, we and others have reported a reduction in dopamine D_2/3_ receptor (D2/3R) binding in the striatum, an important component of the brain reward system [1–3]. The neurotransmitter dopamine is important for the reinforcing value of food and it has been shown that food can induce a release of endogenous dopamine in the striatum [4]. Obese individuals are thought to be more sensitive to food reinforcement than those who are non-obese. This may underlie the notion that obese humans experience an increased craving for food. In addition, striatal D2/3R availability has been linked to craving and diet-induced obesity [5]. Therefore, it is plausible that dopamine-related mechanisms linked to craving and impulsiveness play a role in the development and pathophysiology of obesity. At present, it is unclear whether lower D2/3R availability is a cause or an effect of obesity. If the latter is true, one would expect that the reduced D2/3R availability observed in obese humans is reversed following the loss of a clinically significant fat mass or during a hypoenergetic state.

Obesity is associated with insulin resistance. Insulin receptors are widely expressed in the human brain, and a relationship between insulin sensitivity and central dopamine signalling has been suggested [6]. It needs to be determined, therefore, whether the striatal D2/3R binding potential correlates with hepatic or peripheral insulin sensitivity.

To determine whether the previously reported reduction in D2/3R availability in obese humans is reversible, we studied striatal D2/3R availability before and 6 weeks after Roux-en-Y gastric bypass (RYGB) surgery in 19 morbidly obese women. Eighteen of these patients participated in a study on the short-term metabolic effects of RYGB surgery (NTR1548; for one additional patient only single photon emission computed tomography [SPECT] data could be acquired) [3]. Informed consent was obtained from all participants and the study was approved by the local medical ethics committee of the Academic Medical Center in Amsterdam. We assumed that a difference of 15% in D2/3R availability would be of clinical relevance. A power analysis indicated that we would be able to detect this difference in a study group of 18 individuals. In the morbidly obese women, striatal D2/3R availability was assessed using a brain-dedicated SPECT scanner and [^123^I]iodobenzamide (bolus/constant infusion technique). Acquisition, attenuation correction, reconstruction and analyses of SPECT data were performed as previously described [1]. Apart from a classic region-of-interest (ROI) analysis, data also underwent an MRI-driven analysis. In this additional analysis, SPECT images were co-registered to individual MR images, and ROIs were drawn for the caudate nucleus and putamen separately and for the occipital cortex (representing non-specific binding) on the MR images [7]. Insulin sensitivity was determined before surgery by a two-step hyperinsulinaemic–euglycaemic clamp using a stable glucose isotope tracer [8]. Data for four women were not complete owing to technical failures and were excluded from analysis.

The women had a mean age of 40.4 ± 8 (26–49) years (mean ± SD [range]). Weight loss 6 weeks after RYGB was 14 ± 4.6 (8–24) kg, which resulted in a significant reduction in BMI after surgery (before surgery 45.7 ± 6.3 [38.7–61.3] and after surgery 40.9 ± 6.3 [34.1–57.6] kg/m^2^; *p*  <  0.001). The ROI analysis showed no significant change in D2/3R availability before vs 6 weeks after RYGB (Fig. 1a). Also, in the MRI-driven analysis, the D2/3R availability in the striatum as a whole and in subregions of the striatum (caudate nucleus and putamen) did not change significantly after surgery (Fig. 1b). There was no correlation between BMI and D2/3R availability before (*p*  =  0.35; *r* ^2^ = 0.054) or after surgery (*p*  =  0.51; *r* ^2^ = 0.027). This suggests that striatal D2/3R is not regulated by acute changes in energy balance and is not influenced by fat mass per se.

**Fig. 1:**
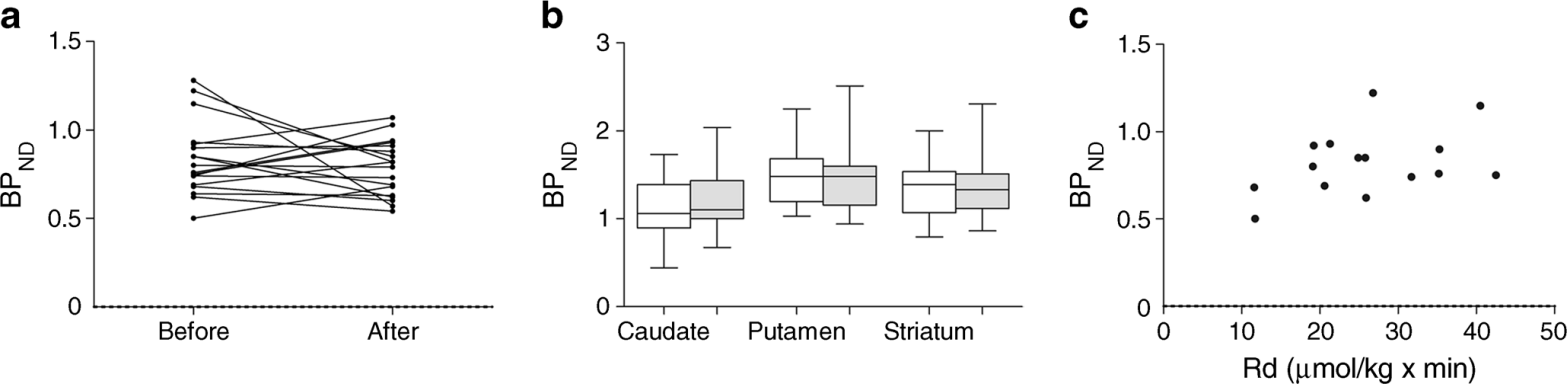
Striatal D2/3R availability of 18 obese women before and 6 weeks after bariatric surgery according to (**a**) classic ROI analysis (without MRI co-registration), mean BP_ND_ before vs after surgery (*p*  =  0.415); (**b**) boxplots showing MRI-driven analysis expressed for the whole striatum as well as striatal subregions (caudate, *p*  =  0.272; putamen, *p*  =  0.661; striatum *p*  =  0.842, before vs after surgery). White bars represent BP_ND_ before RYGB, grey bars represent BP_ND_ after RYGB. (**c**) Striatal D2/3R availability of 16 obese women before bariatric surgery vs insulin-mediated rate of peripheral glucose disappearance (Rd) (*r* ^2^  =  0.156; *p*  =  0.14)

Earlier studies on D2/3R binding after bariatric surgery are contradictory and report either an increase [2] or a decrease [3] in striatal D2/3R availability 6 weeks after surgery. Whether a change in D2/3R occurs after long-term weight loss is unknown. Interestingly, when correlating the pre-surgery level of peripheral insulin sensitivity with pre-surgery striatal D2/3R binding availability, a trend for a positive correlation was observed (Fig. 1c), but there was no correlation with hepatic insulin sensitivity. The former is in line with the general insulin-sensitising effects of dopamine agonists in obese diabetic individuals. In addition, dopamine antagonists are known for their negative effects on insulin sensitivity, and drug-naive schizophrenic patients, known for their disturbed central dopamine metabolism, are characterised by hepatic insulin resistance [9]. Furthermore, a correlation between the insulin sensitivity index (S_I_) and D2/3R availability in the ventral striatum has been reported [10]. This suggests that peripheral glucose uptake, predominantly occurring in skeletal muscle when under hyperinsulinaemic conditions, might be in part regulated by cerebral dopamine metabolism. Although a clear difference in D2/3R availability was found between lean and obese individuals [2], within our obese group no clear correlation between BMI and D2/3R availability was found. This suggests that fat mass per se is not the main determinant of D2/3R availability in obesity. This is in line with the unchanged D2/3R availability, despite clinically significant weight loss. Our study is limited to women only and a potential bias of the menstrual cycle on our outcome cannot be excluded.

In conclusion, surgery-induced weight loss does not significantly increase striatal D2/3R availability in morbidly obese women. This suggests that short-term changes in energy balance in morbidly obese humans do not induce profound alterations in striatal dopaminergic neurotransmission and might predispose obese individuals to regain weight after a hypoenergetic diet. Moreover, the striatal dopamine receptor binding potential is not significantly correlated to hepatic insulin sensitivity but shows a trend for a positive correlation with peripheral insulin sensitivity. This adds to earlier findings on a potential role for cerebral dopamine in glucose metabolism.

## Abbreviations

BP_ND_: Non-displaceable binding potential
D2/3R: Dopamine D_2/3_ receptor
fMRI: Functional magnetic resonance imaging
ROI: Region-of-interest
RYGB: Roux-en-Y gastric bypass surgery
SPECT: Single photon emission computed tomography
